# Monthly Summary

**Published:** 1895-08

**Authors:** 


### Monthly Summary.

FILLING PULPLESS TEETH WITH FISTULOUS OPENING.—In the May number of the *Items of Interest*, Dr. C. N. Johnson, of Chicago, tells how he does this operation. The busy practitioner may not have time to resort to this proceeding, as described by Dr. Johnson. Now let me tell you how to do the operation, and do it quickly.

After getting a direct opening into the pulp chamber, and thoroughly washing out its contents with warm water from a syringe, apply the rubber dam, and dry out the pulp chamber with cotton and hot air, then with Gates-Glidden drills of different sizes, enlarge the canals to the apex; now twist a few shreds of cotton around a Donaldson broach, and saturating in pure carbolic acid and iodoform, use as a piston until the medicament appears at the fistulous opening, and do not cease until it does appear.

Now your tooth is ready to fill; do not wait a day or a week, but go right ahead and fill solidly to the apex. I use shreds of cotton saturated with chlora-percha, and a touch of iodoform, and have yet to see the first case of this kind come back to me for treatment in a practice of fifteen years.—Dr. Agnus D. Cameron in *Dominion Dental Journal.*

---

AFTER several years of observation I am convinced that there is no better method of treating the gums after all deposits are thoroughly removed from the teeth and the use of medicants as may be indicated than friction-massage with the fingers. The daily use of a simple medicated powder, especially at night, before retiring, is very essential, as the putrefactive action of food deposits and acid secretions are more destructive than during the day. Pumice or os-sepia should never be the ingredients of tooth powder to be used daily; neither should a stiff and large brush be used. It is a foolish idea that such a brush is beneficial; both lacerate and cut the gums away, exposing the necks of the teeth to the action of caries and the rapid wearing or notching of the cement. A brush of medium size and stiffness, intelligently used, with a powder containing an ounce of boracic acid to the pound of the usual formula, and an occasional friction-massage will ordinarily insure a healthy mouth. Children should be taught massage of the gums and the use of a brush with an appropriate tooth powder. Absolute cleanliness is also essential to the beauty and preservation of the teeth and health of the mouth. Parents should make an early call on a competent dentist on their behalf for advice and needed attention,—R. J Parrie, Cincinnati, O.

TRANSMISSION OF CHOLERA BY THE HOUSE FLY.—Craig (*Medical Record*) has made some careful experiments by feeding common house flies with a fresh boillon culture cf the cholera spirillum. At the end of three days they seem uninjured, and when the intestinal contents were removed, with every precaution to prevent contamination from the exterior of their bodies, the nature of which was confirmed by culture experiments and the obtaining of the red culture reaction by the addition of acids to beef-tea culture. If the common house fly is able, without injury to itself, to carry and transmit the cause of cholera, its agency in the extention of epidemics is probably important and may be difficult to combat.—*Phila. Polyclinic.*

---

THE PREVENTIVE TREATMENT OF SEASICKNESS.—Chlorobrom, which is a solution of chloralamide and potassium bromide, is the latest remedy proposed for the motion sickness so terrible to many ocean travelers and railway tourists. From the results of its use in 300 cases reported by ship's surgeons in medical charge of steemers plying between Great Britain and the United States, Canada, India, Australia and New Zeland, Dr. M. Charteris, professor of materia medica and therapeutics in the University of Glasgow, reiterates in the *Lancet* of April 21st, that chlorobrom, judiciously taken, will prevent an attack of seasickness, or cut it short if it has already begun. He states, however, that no prophylactic benefit can be secured by the use of chlorobrom in long sea voyages unless for two nights before embarkation, the passenger depletes the stomach and bowels by taking a cholagogue pill. Persons who dread the voyage will be helped by a previous dose of the solution. On board the vessel, the diet should be spare and dry. No full meal should be indulged in, and soup, pastry and sweets especially should be avoided, and a hypnotic dose of chlorobrom should be taken for three nights, 2 tablespoonfuls at a dose for women and a tablespoonful and a half for men. For short voyages, beginning about 10 p. m., the passenger should take one dose of the sizc mentioned and immediately go to bed. Immunito from seasickness is obtained in the great majority of persons if these directions are followed, but if they are not, the result will be that the chlorobrom will have no effect in arresting an attack of vomiting. Teaspoonful doses given every 10 minutes until a tablespoonful or a tablespoonful and a half has been given, will, however, almost invariably check the retching and depression, which Dr. Charteris believes are due to irritation of the vomiting center in the medulla oblongata consequent upon the vomiting caused by the gastric disturbance due to the unusual amount and uniformity of motion.—*New York Medical Journal.*

---

PROTECTING PULPS.—W. Storer How, D. D. S., Philadelphia, Pa., suggests the use of one or two discs of rubber dam as a protection to pulps nearly exposed. The use of a drop of pure mastic varnish in the bottom of cavity will facilitate the placing of disc in position. The second disc may be spread with soft cement and placed cement down on the first, and burnished gently down. Other pulp protectors he suggests, such as thin gutta percha, paraffin paper, vulcanizable rubber, celluloid or mica discs. A piece of oiled mica, he says, makes one of the best matrices to be had, having in its favor thinness, smoothness, flexibility, resistance to acid action, shapability with scissors, to say nothing of cheapness. One of the best ways to fasten such a matrix in place is by using heat-softened gutta percha pressed against it on either side between the teeth.

---

THE APPLICATION OF THE RUBBER DAM.—I have never been able to understand how anything is gained by using small pieces of rubber dam. It should be large enough to well cover the mouth, cheeks and chin, so that it may be held and kept out of the way during operations. Many breaks around the necks of the teeth after the dam has been applied are due to punching the holes too near together. Punch them far enough apart so that the rubber will not be stretched in the interdental spaces, and be sure to punch holes enough. The ligating is easily done by allowing the thread to extend from tooth to tooth on the lingual side without a single knot. Black carpet-thread makes a good ligature, and its color makes it possible to detect any fibres which may be caught on the cervical border of a cavity.—H. Barnes, in *Ohio Dental Journal.*

---

LINING RUBBER PLATE WITH ALUMINUM.—Dr. Thos. Rhodes Pixton, of Philadelphia, gives a method of lining rubber plates, to give metal surface in contact with the mouth. He used aluminum rolled to twenty-eight gauge, annealed and carefully pressed and burnished to cast, using preferably the handle of a tooth-brush for this purpose. By using a small enamel chisel, small loops may be made in the aluminum for attachment of rubber. The teeth are then set up in wax as usual for rubber plate.—*Items of Interest.*

---

VULCANIZING BETWEEN METAL.—For the benefit of Dr. P. Z. Haight, and others, I give my method of vulcanizing between metallic surfaces. Before pouring the second half of the flask burnish pure tinfoil (heavy) on the lingual surface of the wax, letting it cover a part of the grinding surfaces of the teeth, and leave the edge of the tin free so that it will be engaged by the last pouring of plaster.

Before packing the case burnish No. 4 pure tinfoil on the model, and there will be no trouble getting perfect surfaces if the foil is not torn. Do not use anything but pure tin, otherwise there will be dark spots.—Geo. M. C. Barnard in *Items of Interest.*

AMALGAM.—Prof. J. Foster Flagg, in the *International*, says: Has amalgam any good qualities in itself? Have we any good alloys?

How many men know how to use it and get the best results? What curses have been heaped on its hallowed head? Where is the man who dares say he uses it? Anathemas come thick and fast from the M. D.'s of both old and new school, as if they were the Supreme Court to sit on its merits.

Several years ago, at one of the first meetings of our Odontological Society of Pennsylvania, one of its oldest and most prominent members went so far as to place on record in the proceedings: "All men, falling away from manipulative ability, lean on plastics." To-day he is experimenting to find an ideal alloy to help him out in his failures in gold. How sad such a record! Who among you so mean as to deny using it, and without "apologizing?"
I say it is one of the grandest filling materials we have, and while I have spent nearly thirty years in inventing power mallets and special smooth oval points for gold work, I confess I throw all aside very quickly, and while I delight to wield the electric or mechanical mallet in distancing time and space with gold, yet I use more amalgam to-day than ever before in my life.

I am thankful I had the courage and principle to stand by it, and recommend and show how much could be done when condensed under Japanese bibulous paper. To me it is a sheet-anchor for the class of cases every day coming to me from others. If you use amalgam and wish to make your practice a success with it, learn how to manipulate it and prepare your cases for its adaptation.—*Pacific Druggist and Physician*,

---

Dr. A. G. Johnson recommends the following formula for pulp devitalization:

| | | |
|---|---|---|
| R. | Arsenious acid - - - - - | gr. xx. |
| | Hydrochlorate of cocaine - - | gr. xxv. |
| | Lanolin - - - - - | q. s. ft. paste. |

Dr. C. N. Johnston says of small and tortuous canals, that while we may meet many which cannot be drilled out and filled solidly to the apex and yet which remain satisfactory, yet no man is doing his duty who does not make an honest effort to fill all that can be filled by skill and application, and to sterilize as perfectly as possible every canal he cannot fill. In case of a small canal, after reaming out the orifice to insure access, use a fine broach of piano wire to explore as far as possible, then flood with an antiseptic and work up with broach. In trying to fill such canals, some material should be used that is semifluid or entering, should be used so that it may be pumped as far as possible into all spaces.—*Cosmos.*

---

THINKING WITH THE FINGER TIPS.—According to a writer in the Arena the coming man will assuredly perceive and think in every part, from his head down to his feet. He bases this assertion on what, he says, may not be so generally known—that recent post-mortem examinations of the bodies of the blind reveal that in the nerves at the end of the fingers well-defined cells of gray matter had formed, identical in substance and in cell formation with the gray matter of the brain. What, he asks, does this show? "That a man can think, not alone in his head, but all over his body, and especially in the great nerve centres like the solar plexus and the nerve ends on the palms of the hands and the soles of the feet."

---

EROSION.—Nitrate of silver, when applied to an eroded part of a tooth, is decomposed; the oxide of silver—an insoluble and inert substance—is deposited, which protects and relieves hyper-sensitiveness, antagonizes the action of morbific influences, and assists nature to arrest the disease.—Geo. J. Frederichs, in *Pacific Druggist and Physician.*

ANTISEPSIS IN DENTISTRY.—Dr. Shrady, editor of the *Medical Record,* every once in a while indulges in a "fling" at dentists and dentistry. His latest is an accusation as undeserved as usual, at one sweep he accuses the profession of neglecting antisepsis. To one who reads dental literature and attends association meetings of late years, it is surely known that the average dentist pays more attention to cleanliness of instruments and of person than does the average physician. That surely is our own personal experience. If the egotistical editor of the *Medical Record* had taken pains to find the facts we believe he would have arrived at the same conclusion.—*Western Dental Journal.*

---

DECAY OF THE TEETH.—The dentist is confronted with three classes of diseased teeth—those in which the dentine alone is affected; those in which the pulp has been but recently exposed; and those in which, because of prolonged exposure, the pulp is either dead or dying. Of the first class I have already spoken. In a tooth of the second class, the pulp can be cured by the application of soothing medicines, which may remove the irritation and subdue the inflammation, and enable it to bear a filling. If, after being thus treated, the tooth be filled, a covering of secondary dentine may be formed by nature underneath the filling; for the better protection of the pulp, and soon the tooth may become as sound as ever.

Concerning the third class of decayed teeth, there are three kinds. Those in which the pulp has recently died, those in which there is more or less of infection and inflammation, and those with a fistulous opening, with a more or less constant discharge of pus. The first can be cured with comparative ease. The second may be relieved if judicious means are employed, while the third may require a considerable time, and the exercise of much patience and skill on the part of the dentist. The pulp chamber and canals must be thoroughly cleaned and disinfected, and this work is sometimes performed with great difficulty, as the canals are often crooked and difficult of access.

Should the dentist fill such a tooth, leaving within it particles of infected matter in the pulp canal, they may putrify and generate gases, which, having no escape except the opening at the apex of the tooth, press against the surrounding tissues, and produce abscesses and swellings, with their accompanying pains. After the whole territory has been thoroughly disinfected, the canals must be effectually filled. It is sometimes advisable to insert a temporary filling to last for several weeks, and only after this experimental stopping has been sufficiently tried, and no unfavorable symptoms have supervened, should a permanent filling be substituted for the temporary one. If, however, the tooth becomes sore, it is an indication that inflammation is again active, and that unless it be reduced an abscess may follow. The filling must then be removed, and the tedious work of disinfection resumed.

It is apparent, then, that to postpone the work of filling a decayed tooth only increases the danger and suffering, while the result, when accomplished, is much less satisfactory, and much more expensive, painful and uncertain.—*Items of Interest.*

---

EXTRACTING AN ABSCESSED TOOTH.—"A" is suffering from an abscess developed from an irritation produced by a dead tooth. He is about to visit the dentist to have the tooth extracted, when some officious, though well-meaning friend, informs him that it is dangerous to extract the tooth before the abscess has broken, and "A" will suffer many days and nights of intense agony, waiting for the abscess to heal, and after this has taken place, and he no longer suffers torture, he undergoes the additional pain of having the tooth extracted. As a matter of fact, there is no danger in extracting a tooth about which an abscess is developing. There was a time when it was thought hazardous to do so, it is true, but this theory has long since been exploded. Moreover, the pain of extracting such a tooth is entirely lost in the far greater pain suffered from the abscess, for when one suffers from pains of varying intensity, the sensation of the lesser pain is, measurably, lost in that of the greater.—*Items of Interest.*

ROOT CANALS.—Many root canals met with in practice are small, indistinct, and difficult of access, so that they cannot be thoroughly cleared. For many years I have used a ten percent solution of bichloride of mercury in absolute alcohol, applied to the pulp chamber on cotton, wool or other fibre with great success. But since Dr. Miller's paper, read at the Chicago Congress, in 1893, advocating the use of a pellet formed of one-tenth of a grain each of bichloride of mecury and thymol, I have adopted this method in some two hundred cases. but using pellets of only one-fifteenth of a grain, and by far the larger number of cases were very successful. I do not advocate this treatment where the canal could be got at, as I am strongly in favor of filling all root canals where possible, but in almost inaccessible positions the plan suggested is valuable.--*Dental Record.*

---

DIGESTION.—The stomach may be compared to a stove; the food to the fuel consumed by the stove, and life to the heat given off by the glowing coals. The stomach is an excellent stove, and will burn much bad fuel. But have a care lest it rebel, and the fire be extinguished.

Good health demands thorough digestion; thorough digestion demands thorough mastication, and thorough mastication demands sound and healthy teeth. Ulcerated roots and decayed teeth; an inflamed mouth and vitiated saliva, are poorly fitted to supply the stomach 'with food that can be properly digested and assimilated.

Abscesses with agonizing pains, necrosed jaws and probable disfigurement of the face with tumors, and foreign growths, frequently result from a neglected mouth.

Diseases of the eye, ear, and the cavities of the head, often the most difficult to diagnose, may be traced directly to an unhealthy condition of the teeth. But a short time ago, I was visited by a young lady whose eyes were so badly affected that she could only see with difficulty. Medical treatment had failed to relieve her. Having trouble with her teeth, she found it necessary to consult the dentist, and with the curing of her dental troubles her eyesight was restored.

I have seen the most robust patients shattered in health by dental troubles. Who is not familiar with the acute suffering with which the development of an abscess, or swelling on the gums or face, is accompanied? The pain is not only agonizing, but the general health is affected. Surgeons and dentists are daily called on to perform operations for the removal of necrosed portions of bones, and tumors of the most formidable character, and sometimes even for the removal of the entire jaw. There is not a disease to which the human bady is liable that is not aggravated by an unhealthy condition of the teeth.

It is marvelous to observe how men will spend money in the most extravagant manner for outward show, or will wear away the best part of their lives in the accumulation of wealth, and yet never give a thought or a penny to the preservation of health. But there will come a day when disease shall so have wasted their system as to place its recovery beyond all medical skill, and then they will realize the full consequences of their neglect.—*Ex. from Bell's Mouth and Teeth.*

---

ARSENIC.—There are some dentists who promptly apply an arsenical paste to sensitive cavities, whether the pulp be exposed or not, and patients have been led to approve this, thinking thereby to avoid pain during the filling. But it is very bad practice, and the more reputable portion of American dentists condemn it earnestly, and for some of the following reasons:

First. Because when devitalized, there is always the liability to putrefaction and the formation of an abscess.

Second. Because it is much easier to fill a live tooth than a dead one.

Third. Because a dead tooth is liable to many diseases, and is not as permanent or as useful as a live one.—V. C. Bell in *Items of Interest.*

